# Doxycycline-Induced Intracranial Hypertension Presenting as Unilateral Pulsatile Tinnitus

**DOI:** 10.1097/ONO.0000000000000043

**Published:** 2023-11-22

**Authors:** Naushin Shabnam Ali, Barry Daniel Long, Nauman F. Manzoor, Aristides Sismanis, Daniel H. Coelho

**Affiliations:** Department of Otolaryngology—Head and Neck Surgery, Virginia Commonwealth University, Richmond, Virginia

**Keywords:** doxycycline, intracranial hypertension, pulsatile tinnitus, tetracycline

## Abstract

**Background:**

Pulsatile tinnitus (PT) is increasingly recognized as a cardinal symptom of idiopathic intracranial hypertension (IIH). However, clinicians should remain aware of other causes of nonidiopathic or secondary intracranial hypertension manifesting as PT. We present 2 patients with isolated PT (without accompanying headache, blurred vision, and papilledema) thought to be secondary to tetracycline-induced intracranial hypertension. To our knowledge, these are the first cases of PT as the presenting symptom of this condition.

**Cases:**

A 41-year-old female (body mass index [BMI] 29 kg/m^2^) with ocular rosacea was initially treated with minocycline. Shortly after transitioning to oral doxycycline and erythromycin eye ointment, she noted left-sided PT. Her PT resolved after discontinuing doxycycline. In a second case, a 39-year-old female (BMI 19 kg/m^2^) with acne presented with a three-year history of left-sided PT while on long-term oral doxycycline for many years. She denied visual or auditory changes and atypical headaches. MRI findings were concerning for intracranial hypertension. Three months later, the patient was seen by neuro-ophthalmology, with findings suggesting prior papilledema. The patient reported PT improvement after discontinuing doxycycline.

**Conclusions:**

This case series highlights 2 cases of isolated PT as the sole symptom of intracranial hypertension that resolved with tetracycline cessation. The presentation and unexpected improvement following tetracycline discontinuation are atypical compared with previous reports of tetracycline-induced intracranial hypertension. Clinicians should maintain a high index of suspicion for all types of intracranial hypertension (idiopathic and secondary), even in patients with a lower BMI. Current and prior medications should be reviewed when considering the etiology of intracranial hypertension.

Despite increased attention over the last decades, accurate diagnosis of pulsatile tinnitus (PT) etiology remains a challenge. Recent studies have demonstrated a failure to identify a source in up to half of cases (1,2). The differential diagnosis is wide, with dozens of potential sources ranging from anemia and other metabolic derangements to intra- and extra-cranial vascular abnormalities from the chest to the occiput.

One of the most common causes of PT is idiopathic intracranial hypertension (IIH), previously known as pseudotumor cerebri (3). Although the classic presenting triad is headaches and blurred vision (with or without papilledema) in an overweight female of child-bearing age, IIH can have a broad range of clinical presentations (Table 1), with PT becoming increasingly recognized as a common presenting symptom. In fact, in many cases, it can be the only symptom. Moreover, not all intracranial hypertension (IH) is idiopathic and may be due to an underlying cause. What follows are 2 unusual cases of reversible PT as the sole sign of tetracycline-induced IH.

**TABLE 1.** *Various clinical presentations that should raise suspicion for idiopathic intracranial hypertension (IIH) as a possible etiology* (4,5)

IIH Syndrome	Spectrum of clinical presentation
IIH classic:	• Headaches, visual disturbances, papilledema
IIH variants:	• Without papilledema • Presenting mainly with PT • With unilateral papilledema • With minimal symptomology (incidental papilledema) • Presenting with spontaneous CSF leaks (rhinorrhea, otorrhea) • Presenting with temporal lobe epilepsy

CSF, cerebrospinal fluid; IIH, idiopathic intracranial hypertension; PT, Pulsatile tinnitus.

## METHODS

Informed consent for the publication of their clinical information was obtained from all patients.

## CASE PRESENTATIONS

### Patient #1

A 41-year-old white female with a body mass index (BMI) of 29 kg/m^2^ presented to the ophthalmologist with a new-onset eye rash and pain. She was diagnosed with ocular rosacea for which she was initially given minocycline. The patient had significant cramping and diarrhea while on initial therapy and was subsequently transitioned to oral doxycycline and erythromycin eye ointment. She initially began with 25 mg of doxycycline, with planned titration to 50 mg twice a day. Within the first weeks of starting doxycycline, she noted left-sided PT. She denied any hearing loss, vestibular symptoms, vision changes, or headaches different from her typical migraines. She was unable to discern factors that alleviated or worsened her tinnitus, and on physical examination, there was no evidence of papilledema or visual changes on repeat ophthalmologic examination. Nonetheless, the doxycycline was stopped and an MRI was ordered. The patient was then referred to otolaryngology for further evaluation.

By the time she was seen by otolaryngology, she reported that her PT had resolved shortly after cessation of the doxycycline, which had successfully treated her ocular rosacea. The rest of her history and physical examination were unremarkable. The MRI was normal except for a mild focal dilation of the distal right sigmoid sinus, contralateral to her perceived PT. Further imaging with temporal bone CT showed contour deformity of the right mastoid air cells without dehiscence into the mastoid or tympanic cavities, making developmental diverticulum much more likely than an acute abnormality of the right sigmoid sinus. The putative diagnosis, established and agreed upon by the involved specialists, was temporary IH secondary to doxycycline use.

### Patient #2

A 39-year-old white female with a BMI of 19 kg/m^2^ presented to otolaryngology with a 3-year history of left-sided PT. She had a longstanding history of acne for which she had been on oral doxycycline 100 mg twice a day for several years. She denied vision changes, hearing loss, autophony, sound- or pressure-induced dizziness, or headaches different from her usual migraines. Physical examinations, including otoscopy, and audiologic testing were unremarkable.

MRI was obtained demonstrating focal stenosis of the distal aspect of the bilateral transverse sinuses. Empty sella and borderline low-lying cerebellar tonsils were also noted. Given imaging findings concerning IH, a referral was placed to neuro-ophthalmology.

When seen by neuro-ophthalmology 3 months later, there was no evidence of papilledema on physical examination. A high watermark was noted on fundus photography suggestive of prior papilledema. At that visit, the patient disclosed that shortly after being seen by otolaryngology, she stopped taking her doxycycline prescription as it was suggested that doxycycline may be associated with IH and therefore PT. She noticed an improvement in her PT since stopping her doxycycline. At that point, the working diagnosis established and agreed upon by the involved specialists was temporary IH, now resolved, secondary to chronic doxycycline use.

## DISCUSSION

The most common form of IH is IIH, defined as increased intracranial pressure in the absence of mass lesion, infection, or abnormalities of brain parenchyma. This condition is familiar to otolaryngologists with many potential clinical manifestations in the head and neck (3,6–8). The vast majority of patients with this condition share common demographic characteristics (female, obese, child-bearing age) (9). However, and perhaps less well known to otolaryngologists, not all IH is idiopathic and can present secondary to cerebral venous stenosis, Grave’s disease, or many medications including tetracyclines, corticosteroids, or isotretinoin (10,11). Chief among the studied medication classes is the tetracycline antibiotics, especially minocycline and doxycycline. They have long been associated with the precipitation of IH and are often prescribed to patients for acne vulgaris or other skin conditions.

The second-generation tetracyclines, doxycycline and minocycline, are broad-spectrum antibiotics used for a variety of conditions such as ocular rosacea, Lyme disease, malaria prophylaxis, and acne vulgaris. Multiple cases have been published documenting the association of new-onset IH with first- and second-generation tetracyclines, with the strongest association with minocycline (12). Symptom onset ranges from 2 weeks to greater than 6 months following drug initiation. These patients most commonly present with a complaint of new-onset headaches or transient visual obscurations or diplopia, as well as nausea or vomiting (10,11).

The mechanism by which tetracyclines cause IH is not yet understood, though one theory posits antibiotic interference with cerebrospinal fluid (CSF) absorption mechanisms at the site of arachnoid granulation (13). Minocycline has the highest lipophilicity of the tetracyclines, which allows increased penetration of the blood-brain barrier (14). As a result, several isolated vestibular adverse effects have also been reported with minocycline, such as headache, dizziness, vertigo, and tinnitus (15). Interestingly, while multiple cases have been reported that attribute doxycycline use to IH (16–18), isolated vestibular adverse events were only reported with minocycline (15,19,20).

The unique aspects of these cases are twofold. First, these patients presented with isolated PT rather than any of the common triad of headache, visual changes, and papilledema as seen in the previously reported cases (10,11,15). While the association between IIH and PT has been well documented, the relationship between PT and secondary IH is less clear. Sismanis reported his experience with 82 patients with PT and IH, of whom 10 were secondary IH (21). Although the exact pathophysiology of PT in IH remains incompletely understood, this case series may highlight differences in PT physiology between primary (idiopathic) and secondary IH. The vast majority of published research on IH and PT is in IIH. Perhaps a closer inspection of PT in secondary IH will shed additional light.

Second, all previously reported cases had persistent symptoms of IH even after tetracyclines were stopped—with many requiring medical or procedural intervention to address the signs and symptoms of IH (18,22,23). Two of the reported cases required a ventriculoperitoneal shunt for symptom control, another needed optic nerve decompression, and others had chronic, stable vision loss. In other instances, patients require long-term acetazolamide treatment, which helps to decrease CSF production, or lumbar punctures to relieve pressure.

This case series aims to add to a growing body of evidence that shows a link between tetracyclines and the development of IH but also presents an illustrative example of early diagnosis and symptom resolution without a need for any intervention other than the cessation of the offending agent. As otolaryngologists are becoming more aware of both the workup of PT and the clinical manifestations of IIH, we must also remember that not all cases of IH are indeed idiopathic. Given the prevalence of both conditions, it is likely that PT as a presenting symptom of IH is underrecognized and underreported. Clinicians must remember to review medications thoroughly (even those that have been stopped) and to entertain the possibility of any other causes of IH even when a patient’s demographics might suggest IIH.

## FUNDING SOURCES

None declared.

## CONFLICT OF INTEREST STATEMENT

None declared.

## Data Availability

Data sharing is not applicable to this article as no datasets were generated or analyzed during the current study.
